# Challenges in Establishing Vaccine Induced Herd Immunity through Age Specific Community Vaccinations

**DOI:** 10.14336/AD.2021.0611

**Published:** 2022-02-01

**Authors:** Barsha Dassarma, Satyajit Tripathy, Matimbha Chabalala, Motlalepula Gilbert Matsabisa

**Affiliations:** Department of Pharmacology, School of Clinical Medicine, Faculty of Health Sciences, University of the Free State, Bloemfontein9300, SA

**Keywords:** Coronavirus disease 2019 (COVID-19), COVID-19 vaccines, Herd immunity, indirect immunization

## Abstract

Presently, the second wave of COVID-19 pandemic is driving the world towards a devastating total failure of the healthcare system. The purpose of the review is to search for the studies reporting on the implication of herd immunity into a naïve population through age specific mass vaccination. This review is based on selected publications on the effect herd immunity to COVID 19 in communities. We searched published scientific articles, review articles, reports, published in 2020 as well as read some basic, cult publications related to establishment of indirect immunity to a population. We have focused on use of application of vaccine induced herd immunity into community to confer indirect immunity against COVID-19 and searched on electronic databases, including PubMed (http://www.pubmed.com), Scopus (http://www.scopus.com), Google Scholar (http://www.scholar.google.com), Web of Science (www.webofscience.com) and Science Direct by using key words such as Herd immunity, indirect or passive immunization, Coronavirus disease 2019 (COVID-19), severe acute respiratory syndrome, coronavirus 2 (SARS-CoV-2), and immune-technique. This review proposes the implication of mass vaccination-induced herd immunity in a population to curb the infection, and to every individual in a given population irrespective of their age.

Currently, the world is faced with new pandemic, COVID-19 disease, now together with all the ever-emerging variants of the corona virus. The worldwide spiraling reported new cases (172,630,637 cases as of June 6, 2021) of COVID-19 posing direct challenges to public healthcare and infrastructures and is becoming a grave threat to the world populations (https://covid19.who.int/?gclid=CjwKCAjw5Ij2BRBdEiwA0Frc9dhAQsQCFXEF4dGkq3FlP98JlSUP-oyXoo0lYI_bpa1v87goEhmhDxoCJb4QAvD_BwE). The situation is worsened, as the disease mode of transmission is now known to be through a vast number of asymptomatic and pre-symptomatic persons and including the emergence of new viral variants [1]. The COVID-19 disease is becoming one of the worst pandemics in the history of mankind. The worldwide overall mortality rate reached up to 4.5% as number of deaths crossed 600,000 deaths out of 13 million COVID-19 cases [2]. For young adults without comorbidities, the death rate has been below 0.2% whereas, for the elderly population, the death rate due COVID-19 is said to reach up to 15% [2]. The recovery rate varies with age, health as well as with severity of the disease. Majority of infected patients of the COVID-19 experience cough, fever, headache, loss of smell and taste, shortness of breath and fatigue. Severely infected patients develop acute respiratory distress syndrome (ARDS) in association with a huge cytokine storm within 2- 14 days of post viral infection [3]. The application of corticosteroids as well as low dose dexamethasone drugs against COVID-19 has become controversial due to their delayed renal clearance [4]. The application of Remdesivir and Lopinavir/ ritonavir against COVID-19, have generated much controversy around their efficacy and hence the use of the said has not been generally supported [5]. Discovery of vaccines against COVID-19 is a superior achievement by advanced molecular and biological science. As of 6^th^ June 2021, a total of 2,121,290,083 vaccine doses have been administered to patients worldwide (www.google.com/search?q=covid+19+vaccination+statistics+world+wide&rlz=1C1GGRV_enZA891ZA891&oq=covid+19+vaccination+statistics+world+wide&aqs=chrome..69i57j0i13j0i390l2.12815j0j7&sourceid=chrome&ie=UTF-8) (Table.1.). This review will deal not only with the chances of eradication of the disease through vaccine induced herd immunity in a population but also with the limitations of applications of the said.

**Table 1:** Worldwide COVID-19 Vaccination Statistics.

Country	Doses Given	Fully Vaccinated	Percentage of Population Fully Vaccinated
United States	304M	140M	42.8%
India	234M	45.3M	3.3%
Brazil	74.5M	23.5M	11.1%
France	40.8M	12.9M	19.2%
Turkey	31.6M	13.4M	16.3%
Russia	31.3M	13.5M	9.4%
United Kingdom	68.8M	28.2M	42.4%
Italy	39.3M	13.3M	22.1%
Spain	30.5M	11.3M	24.0%
Germany	55.5M	18.2M	21.9%
South Africa	1.43K	481K	0.8%
Canada	26.9M	3.18M	8.5%
Iran	4.55M	638K	0.8%

(Source: https://www.google.com/search?q=covid+19+vaccination+statistics+world+wide&rlz=1C1GGRV_enZA891ZA891&oq=covid+19+vaccination+statistics+world+wide&aqs=chrome..69i57j0i13j0i390l2.12815j0j7&sourceid=chrome&ie=UTF-8)

## Study Design and Methodology

This review mathematically establishes the rate of success in the application of “herd immunity” in a community against COVID-19. We have gathered information from Electronic databases which includes PubMed (www.pubmed.com), Scopus (/www.scopus.com), Google Scholar (www.scholar.google.com), Web of Science (www.webofscience.com), Science Direct and website of World Health organization (www.who.int/emergencies/diseases/novel-coronavirus-2019). We have used key words such as herd immunity, passive immunization, Vaccine against Covid-19, coronavirus disease 2019 (COVID-19), as well as severe acute respiratory syndrome coronavirus 2 (SARS-CoV-2).

## Vaccine Induced Herd Immunity: The Concept

Herd immunity is an ancient concept where immunity is said to confer an indirect protection to a susceptible individual against a specific pathogen in a certain population. According to the concept of herd immunity, individuals become immune either by recovering from an earlier infection or by vaccination [6]. The concept of herd immunity can only be applied for the purpose of eradication of a contagious disease, which is transmitted from one individual to another [7]. For example, Tetanus is an infectious disease but not contagious, therefore herd immunity would not work in this instance.

In a given population, a novel pathogen propagates through vulnerable individuals in an uncontrolled manner and therefore increasing the number of infected individuals. On the other hand, if any fraction of the population gets immune to that same pathogen, the probability of being infected is reduced even after a close contact between vulnerable and normal individuals. For many individuals, in a naive population, becoming immune against the pathogen reduces significantly the transmission rate of the pathogen. With the spread rate of the pathogen in a naive population being reduced, the prevalence rate will subsequently decline [8]. Therefore, herd immunity protects a given population or community by suppressing the transmission of the infectious pathogen and subsequently the spread of the disease [9].

**Figure 1.**
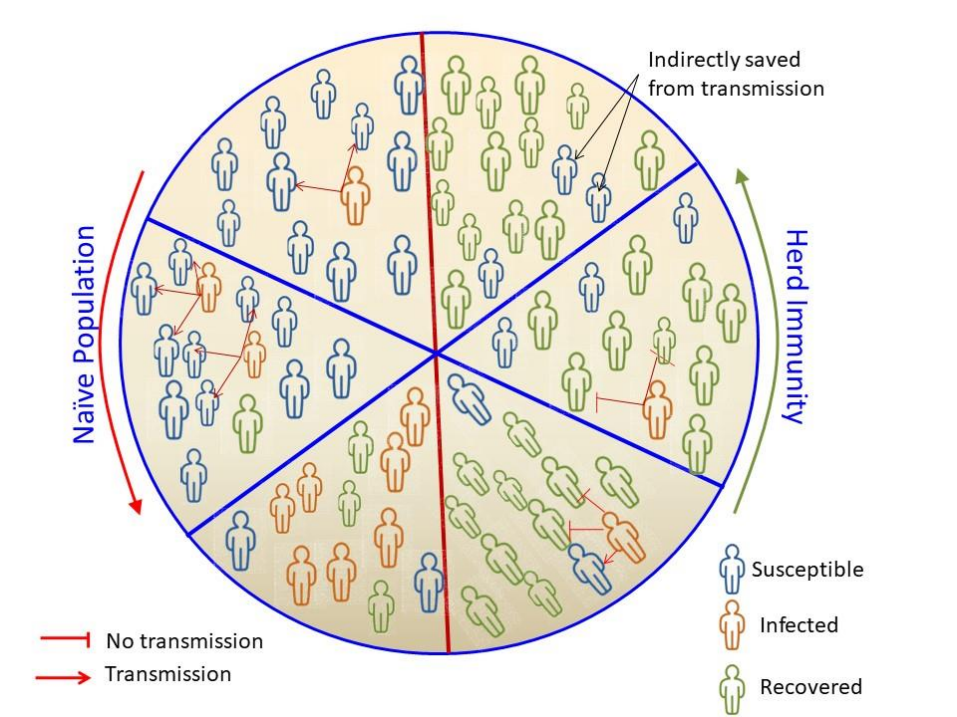
Schematic Representation of Implication of Herd Immunity in a Population.

Herd immunity threshold is defined as a particular point in each population, when the percentage of susceptible individuals lies below the minimum percentage of individuals needed for the successful transmission of the pathogen [10]. The percentage of the population infected when it is above the herd immunity threshold value would only be beneficial with indirect protection from pathogen [10] (Fig. 1). For an eventual gradual disappearance of a disease in a population, herd immunity threshold should be reached [7]. This form of elimination of disease, when there is a permanent reduction of the number of infections toward zero world- wide infections, is called eradication of the disease [11].

The easiest implementation of herd immunity in a population requires adequate amount of vaccination. In the 1930’s, it was first observed that, children became immune against measles because of decreased infection rate even into an unvaccinated population [12]. Since this observation, long mass vaccination has been used to induce herd immunity in a population to reduce the spread of infectious disease [13]. Also, research reports from Kim et al., 2011, showed that vaccine induced herd immunity from one age group may confer protection to other age groups as well [14]. According to several reports, community vaccination against pertussis in adults is also found able to confer immunity to young persons and infants [15, 16]. It has also been seen in the case of community vaccination against *Pneumococcus* disease and *Rota* virus diseases where vaccinated individuals were able to confer immunity toward their unvaccinated siblings [17]. But, with the increased age and waning immune system, the efficacy of the vaccine is decreased [18].

## Vaccine induced Herd Immunity, a “Persona Grata” in COVID-19 Treatment

If a vaccine induced herd immunity can be established in a naïve population against any specific pathogen for a sufficient time, the disease may be inevitably eliminated with zero rate of transmission and the disease may also be declared eradicated [7]. To date, diseases as if rinderpest and smallpox are successful examples of diseases eradication from naïve population through establishment of vaccination induced herd immunity [19]. Mandatory vaccination may be beneficial for the implementation of vaccine induced herd immunity in naive populations [20].

According to the mathematical model for herd immunity as described by Anderson and May in 1985, herd immunity threshold depends on the basic reproduction number of a population (R_0_). The basic reproduction number refers to the generation of secondary cases by a single infectious individual at the starting point of a novel outbreak when the rest of the population is susceptible [9]. Consequently, the larger the value of R_0_, the higher the risk of communicability of the pathogen into a general population [10].

In a naive population, for example where the R_0_ is 4, means the novel pathogen may have the ability to infect at least 4 individuals from a single infected host throughout the infectious period. These 4 cases will spread the infection to 16 new individuals in the population during the infectious period. The herd immunity threshold may be depicted mathematically, as

1-1/R_0_
=1-1/4
=3/4

Therefore, the herd immunity threshold for the population against the novel outbreak is 0.75. This means only 3/4 of the population will be immune to the pathogen. According to Wu et al., 2020, the value of R_0_ for COVID-19 is 2.68 [2]. In a naïve population, from the value of R_0_, the minimum percentage of population (Y) required to achieve herd immunity can be determined [7].

Y= [(R_0_-1)/R_0_] X 100
= [(2.68-1)/2.68] X 100
=62.686

As a result, in a naïve population, for a R_0_ value of 2.68, a minimum of 62.686% of the population needs to be immune to gain herd immunity against COVID-19. With this R_0_ value of COVID-19 which lies between 2.5- 3.5, it will require 60%- 72% of population to vaccinate to confer herd immunity to the whole population [21]. Therefore, to establish vaccine-induced herd immunity for purpose of suppression from a community about 63% of the population needs to be vaccinated. If any population with a R_0_ value of 2.68 get succeed to achieve the percentage, then the rate of the spreading of the disease will be zero. Therefore, the daily rate of vaccination should be increased. Apart from vaccination, to avoid getting new COVID cases, individuals in a population should abide by self-protection rules, maintain social distancing and restrict travel so that rate of spreading of the disease becomes slow.

To control this pandemic at its earliest, recovered individuals could contribute plasma to be used in infected person to subsequently offer immunity into the population. Joseph et al, has further nullified the chance of reinfection by novel Corona virus. According to Joseph G, redetection of the Corona virus may be as the result of diagnostic error or may be just the reactivation of latent COVID-19 [22]. Herd immunity cannot act as the only long-term alternative option to provide immunity from a specific pathogen in a population.

Though herd immunity may act as an indirect way to provide immunity from a pathogen in a population, but it can’t act as the only long-term option to confer immunity against COVID-19 in a population. In addition, the emergence of a new virus strain, due to mutation, may raise a challenge to this indirect population immunity. As one of the currently available options, in the absence of any vaccines or chemotherapeutics against COVID-19, the concept of herd immunity may be applicable to confer indirect immunity in a given population.

## Obstacles for Implementation of Vaccine Induced Herd Immunity against COVID-19

The implementation of vaccine induced herd immunity may confer an immediate indirect immunological protection in a given population by slowing or halting the spread of the disease in a community. Herd immunity is an age-old immune-technique that may be an important alternative intervention to control current covid-19 pandemic. Herd immunity as a method of providing immunity has always been under radar because of conditions like immunodeficiency or immunosuppression that may not always confer immunity to the individual [23, 7].

Application of herd immunity in a population may be found challengeable if number of free rider increases [24]. Free riders in a population are unvaccinated people in a population who benefit from immunity development because of those who have been vaccinated and probably paid for the vaccination. There is always a disproportionate relationship between free riders and a successful establishment of herd immunity. Individuals may choose to free riders for a variety of reasons including a belief that may be vaccines are not effective [25]. In addition, the risks associated with vaccines sometimes excludes individual and give rise to number of free riders [25]. The main obstacle to establishing ring vaccination induced herd immunity in a population is increased number of free riders in the same population. Inadequate vaccination rate in a given population may lead to return of the disease into the population. According to Rackel and Rackel, a proportion from a vaccinated population may also not develop long-term immunity against the pathogen [26].

It should be noted that vaccination methods for the implication of herd immunity should be country-specific. To eradicate the infection, it would be ideal for the countries with enough resources and smaller population (like; countries of European states) to target for total vaccination. In the contrary, countries with large populations may not be able to achieve the whole population vaccination goal due to financial and logistic challenges. Developing countries with huge population burden may face deceased health economy due to pandemic, resource crunch, poor management system to establish mass vaccination in a naive population. To overcome such factors countries with huge populations may choose “vaccination of vulnerable sections” (e.g. aged people, health workers, persons with comorbidities) first. Afterward, they may opt for “age-specific vaccination” (with different age groups) followed by ring vaccination as an alternative vaccination method [27].

Differences in population density, population age structure, comorbidity rates, behavioral patterns, and the implementation of a consistent R_0_ across the world may prove a challenge and make herd immunity not a reliable treatment intervention [2]. Geographical variations, as well as cultural differences, may also contribute to different transmission rates of the disease across the world. Varying population densities across the world, the viral loads of the disease may differ. In addition, gaining immune power also differs between individuals in the same population. Furthermore, to achieve herd immunity into a naïve population requires a large fraction of the population to be infected, which may lead to a devastated situation for any country. Despite all these challenges as presented, the potential benefit of trying and using herd immunity may be the option available to fight the pandemic.

## Molecular Evolution as an Antagonist of Ring Vaccination Induced Herd Immunity

According to the nature of evolution, evolutionary pressure provokes pathogen to undergo constant mutations. Herd immunity itself acts as a stimulatory factor for further mutation of the pathogen. This kind of evolutionary pressure encourages the microorganism to mutate and produce a novel strain [28]. Eventually, this mutant novel strain will be able to evade the herd immunity and therefore proceed to infect people. Virus uses antigenic drift to produce novel strains to escape from vaccination induced immunity and imposes selection pressure into the community. The recommended vaccines against SARA-COV-2 are currently based on a version of spike glycoprotein. Studies with genome sequencing of SARS-CoV-2 show a nucleotide substitution rate of roughly 1×10^-3^ substitutions per year which showed a similar rate of producing mutants of the Ebola virus (1.42×10^-3^) [29,30]. SARS-CoV-2 may use single point mutation, recombination, insertions, and deletions to produce mutants [31]. The rapid rate of mutation may produce multiple genotypic variants but still, vaccines may successfully act, as an immune- booster because old versions spike glycoproteins would generate protective antibodies against newer emerging variants [32].

## Antibody-Dependent Enhancement (ADE) and Vaccine induced Herd Immunity

One of the potential factors to account for is the risk of development of Antibody-Dependent Enhancement (ADE) in individual’s post-vaccination. Antibody-based vaccines while conferring immune-boosting activity may result in ADE. ADE may be triggered by excessive immune complex formation or by enhancing Fc mediated effector functions, which enhances inflammation. These vaccines may result in enhanced antibody-mediated virus uptake which leads to increased replication of the viral body thus enhance infection [33]. Studies showed that ADE may contribute to higher antibody titers which eventually enhances viral load [34]. Increased *in vivo* viral load may trigger cytokine storms further. During the development of immune-boosting COVID vaccines, ADE had been observed in animal studies but a clinical trial with humans did not show any pieces of evidence of ADE post vaccination [35]. Though ADE is a theoretical possibility associated with antibody-based vaccines it may be avoidable (e.g. individuals are requested not to vaccinate themselves who didn’t pass three months after being COVID positive once).

**Table 2:** Global Infection Fatality Rate (IFR) of COVID-19 Among Different Age Groups (Source: Ref no. 36).

Age Groups	IFR
0-34	0.004%
35-44	0.068%
45-54	0.23%
55-64	0.75%
65-74	2.5%
75-84	8.5%
85+	28.3%

## Immunosenescence and Vaccination

The term immunosenescence refers to gradual deterioration of the immune system with advancement of age. A systemic meta-data analytic study in 2020 showed significant differences in COVID-19 infection fatality rate (IFR) among different age groups. Meta-regression estimate of IFR has been found low for children and young adults (e.g., 0.002% at age of 10, 0.01% at the age of 25) whereas a progressively increased pattern for IFR with age has been noted (Table 2.) [36]. Also, older adults are found to be at greater risk for requiring hospitalization or death.

With age complications like acute respiratory distress, septic shock, multi-organ failure, cardiac arrest, arrhythmias, heart inflammation was increased [37]. Increased age contributes to decreased antibody production. Time taken by the elderly for neutralization of viruses is longer compared to young individuals, which lead to an increase in *in vivo* viral load. With age, the functional capacity of T cells is mostly influenced. Due to Immunosenescence, older people face a reduction in the number of naïve T cells available to respond to a vaccine. With age, CD4+ T cell development gets impaired. Impaired development of CD4+ T follicular helper cells hampers the maturation of B cells, which eventually lowers antibody production [38]. Senescence reduces the expression of specific proteins thus antibodies functions get restricted [39]. Additionally, significant decrease in CD8 T cells with age and alteration of the normal ratio of CD4:CD8 have also been found in elderly. Which eventually leads to hampered immune defenses against viral pathogens especially cytotoxic CD8+ T cells [40].

Furthermore, *in vivo* cytokine storm had been pointed out as a prime factor for elderly people to combat with. Due o an increase in serum levels of Interleukin-1 (specifically IL-1B), Interleukin-2 (IL-2), Interleukin-6 (IL-6), Interleukin-10 (IL-10), tumor necrosis factor- α (TNF- α), Interferon- γ (IFN- γ) complications like respiratory distress syndrome, several blood clotting events, vasculitis, acute kidney injury got associated with COVID-19 [41]. Moreover, increased proinflammatory cytokines may affect microglia, neurons as well as astrocytes [41]. Due to the weakening of the immune system older people are taking more time to neutralize the virus and by this time viral load increases. The older population across the world becoming vulnerable to COVID-19. For this reason, the older population has become a priority to be vaccinated against COVID-19. But, the efficacy of the vaccine may vary according to specific *in vivo* repercussions.

Therefore, the older community may become able to vaccine early than other age groups, but its immune-boosting function remains questionable. The vaccine may become less effective to boost cellular immune responses compared to other age groups. Furthermore, the consequences and side effects of an immune-boosting vaccine on older people may also be even more uncertain.

## Conclusion

The application of several sanitization habits, maintaining social distancing, quarantine, complete lockdown of societies, wearing personal protecting equipment, all seem not to have produced the desired and intended effects to control the COVID-19 pandemic as no eminent flattening of the curve is seen. For many developing countries, the implication of ring vaccination induced herd immunity has been found as a cost-effective measure for the eradication of a contagious disease from a naive population. Though vaccines are the best solution available worldwide to develop immunity against COVID-19, the immune-boosting capacity of vaccines don’t last for a lifetime and their effectiveness may change with each mutated strain of the virus. To overcome such fallacies repeated vaccination within a maximum duration of six months might be effective. Age-specific vaccination may provide some boost to an individual’s immune responses, which can cease the rapid and devastating spread of the disease. But for the speedy eradication of the worldwide pandemic situation vaccination must opt for every single individual in a population irrespective of their age. Vaccination should be for all. This type of indirect immunization, may, for now, act as one of the possible ways to confer immunity to naïve populations in the absence of vaccines and pharmaceuticals against COVID-19.
